# Clinician and Patient Perspectives in Oral Lichen Planus: Correlation and Drivers of Disease Severity and QOL

**DOI:** 10.1016/j.xjidi.2025.100424

**Published:** 2025-10-17

**Authors:** Emma L. Myers, Sarah G. McAlpine, Donna A. Culton

**Affiliations:** 1 University of North Carolina School of Medicine, Chapel Hill, North Carolina, USA; 2 Department of Dermatology, University of North Carolina, Chapel Hill, North Carolina, USA

**Keywords:** Disease severity, Oral lichen planus, Oral mucosal disease, Patient-reported outcomes, QOL

## Abstract

Oral lichen planus is a chronic, immune-mediated inflammatory condition that significantly impacts QOL. Clinician-reported measures, such as the Oral Disease Severity Score (ODSS), systematically evaluate clinical severity but may not adequately reflect patient experience. Patient-reported outcome measures, such as the Chronic Oral Mucosal Disease Questionnaire (COMDQ-26), provide QOL insights, although their relationship with clinical severity remains underexplored. This study aimed to assess alignment between clinician-assessed severity (measured by ODSS) and patient-reported QOL (measured by COMDQ-26) in oral lichen planus across sex and disease subtypes as well as to identify key drivers of these scores. A retrospective review of 78 patients with oral lichen planus was conducted with assessment of demographics, disease subtypes, ODSS, COMDQ-26, and pain scores. Erythematous and erosive/ulcerative subtypes had the highest severity and QOL impact scores, with no significant score differences between these subtypes. ODSS components (site, activity, pain) contributed equally to total severity, whereas the COMDQ-26 patient support domain most strongly drove QOL scores. Moderate ODSS–COMDQ-26 correlations indicated only partial alignment between clinician- and patient-reported measures. We conclude that integrating patient-reported outcome measures into clinical practice and trials may improve oral lichen planus care through more comprehensive, individualized, and subtype-focused strategies.

## Introduction

Oral lichen planus (OLP) is a chronic, immune-mediated inflammatory disorder of the oral mucosa, affecting approximately 1–2% of the global population, with a marked predominance among middle-aged women (Al-Hashimi et al, 2007). Clinically, OLP is characterized by white striations, erythematous plaques, and/or erosive/ulcerative lesions, which are often painful and significantly impair patients' QOL by disrupting essential functions such as eating, speaking, and overall psychological well-being (Eisen, 2002; Lozada-Nur and Miranda, 1997). As a T-cell–mediated autoimmune condition targeting basal epithelial cells, OLP presents a complex challenge for both clinicians and patients owing to its chronicity, relapsing nature, and lack of a definitive cure (Eisen, 2002).

Effective management of OLP requires a comprehensive understanding of both clinical disease severity and the patient’s subjective experience. Traditionally, clinical assessment has relied on physician-reported outcome measures, such as the Oral Disease Severity Score (ODSS), which provides standardized and objective evaluations of disease activity and lesion severity (Ormond et al, 2022). Although these metrics are indispensable for monitoring clinical parameters, they may fail to capture the full impact of OLP on patients’ daily lives and emotional well-being (Ormond et al, 2022; Ní Ríordáin and McCreary, 2010; Ní Ríordáin et al, 2015).

Patient-reported outcome measures, such as the Chronic Oral Mucosal Disease Questionnaire (COMDQ-26), address this limitation by evaluating QOL through key domains, including pain, psychological distress, functional impairment, and social isolation, providing a more nuanced view of the patient experience (Ormond et al, 2022; Ní Ríordáin and McCreary, 2010; Ní Ríordáin et al, 2015). Previous research has demonstrated that QOL measures, such as COMDQ-26, capture aspects of disease burden that may not be fully reflected in clinical assessments (Ní Ríordáin and McCreary, 2010). However, although clinician- and patient-reported measures are both widely used, few studies have systematically compared their alignment in OLP (Hashemipour et al, 2024; Wang and van der Waal, 2015). Understanding how these tools correlate—and where they diverge—can help optimize disease assessment and improve individualized treatment strategies.

Moreover, OLP subtype classification and sex-related differences remain poorly understood. Although clinical severity classifications distinguish between reticular, erythematous, and erosive/ulcerative subtypes, the extent to which these correlate with patient-experienced burden remains unclear. In addition, current evidence suggests that sex may influence disease perception and impact, further underscoring the need for a multidimensional assessment approach (Dvorak et al, 2015).

To address these gaps, this study investigated the alignment between clinician-reported severity (as measured by ODSS) and patient-reported QOL (as measured by COMDQ-26) in individuals with OLP as well as the key drivers of these scores. We hypothesized that disparities exist, particularly in psychosocial domains, and aimed to identify the factors contributing to these discrepancies. Our findings provide insights into the multifaceted impact of OLP on patients, offering strategies to enhance patient-centered care through tailored disease management. By identifying the relationship between ODSS and COMDQ-26, this study may impact how disease severity and QOL are incorporated in management decisions in clinical practice and in clinical trial design.

## Results

### Patient characteristics

The study included 78 patients with OLP, with an average age of 67.8 years. Much of the cohort was female (85.9%) and White (91.0%), with limited representation of other racial groups (1.3% African American, 7.7% not recorded). The most common OLP subtype was erosive/ulcerative (62.8%), followed by erythematous (11.5%) and reticular (7.7%), with subtype data not recorded/reported for 17.9% of patients. Concurrent nonoral involvement was observed in 38.5% of patients, with cutaneous (16.7%) and vulvovaginal (15.4%) sites being the most frequent. Nail involvement was not observed (Table 1).

**Table 1:** Patient Demographics and Clinical Characteristics

Characteristics	All Patients with OLP	Reticular OLP	Erythematous OLP	Erosive/Ulcerative OLP	Subtype Not Specified
Total number of patients	78	n = 6, 7.7%	n = 9, 11.5%	n = 49, 62.8%	n = 14, 17.9%
Age (average), y	67.8	68.5	66.8	67.0	
Sex					
Female	n = 67, 85.9%	n = 5, 83.3%	n = 9, 100.0%	n = 43, 87.8%	n = 10, 71.4%
Male	n = 11, 14.1%	n = 1, 16.7%	n = 0, 0%	n = 6, 12.2%	n = 4, 28.6%
Race					
African American	n =1, 1.3%	n = 0, 0%	n = 0, 0%	n = 1, 2.0%	n = 0, 0%
Asian	n = 0, 0%	n = 0, 0%	n = 0, 0%	n = 0, 0%	n = 0, 0%
Not recorded	n = 6, 7.7%	n = 0, 0%	n = 2, 22.2%	n = 4, 8.2%	n = 0, 0%
White	n =71, 91.0%	n = 6, 100.0%	n = 7, 77.8%	n = 44, 89.8%	n = 14, 100.0%
Sites of additional involvement					
Cutaneous	n = 13, 16.7%	n = 0, 0%	n = 3, 33.3%	n = 9, 18.4%	n = 1, 7.1%
Esophageal	n = 3, 3.8%	n = 1, 16.7%	n = 0, 0%	n = 2, 4.1%	n = 0, 0%
Scalp	n = 3, 3.8%	n = 1, 16.7%	n = 1, 11.1%	n = 1, 2.0%	n = 0, 0%
Nail	n = 0, 0%	n = 0, 0%	n = 0, 0%	n = 0, 0%	n = 0, 0%
Ocular	n = 2, 2.6%	n = 0, 0%	n = 0, 0%	n = 1, 2.0%	n = 1, 7.1%
Scalp	n = 3, 3.8%	n = 1, 16.7%	n = 1, 11.1%	n = 1, 2.0%	n = 0, 0%
Vulvovaginal	n = 12, 15.4%	n = 2, 33.3%	n = 2, 22.2%	n = 8, 16.3%	n = 0, 0%

Abbreviation: OLP, oral lichen planus.

Presented is a summary of the demographics, sex, race, and additional sites of involvement of 78 patients with OLP, categorized by subtype.

### Disease severity and QOL measures

#### Overall ODSS and COMDQ-26 scores

The mean total ODSS score was 20.1 (SD = 13.1) of 106, with subsection averages of 6.3 (SD = 3.7) for site, 10.8 (SD = 8.6) for activity, and 3.0 (SD = 2.3) for patient-reported pain (Table 2). The mean total COMDQ-26 score was 41.6 (SD = 18.8) of 104. Domain-specific averages were as follows: pain and functional limitation, 14.0 of 36; medication and treatment, 10.4 of 24; social and emotional, 11.7 of 28; and patient support, 5.2 of 16 (Table 3).

**Table 2:** ODSS Scores in Patients with OLP

ODSS Parameter	All Patients with OLP			Reticular OLP		Erythematous OLP		Erosive/Ulcerative OLP	
	Mean	SD	Maximum Score	Mean	SD	Mean	SD	Mean	SD
ODSS total score	20.1	13.1	106.0	11.8	7.0	19.5	8.0	23.6	14.1
ODSS total among male patients^1^ (n = 11)	11.0	10.8	106.0	6.0	N/A	N/A	N/A	14.4	12.9
ODSS total among female patients (n = 67)	21.4	12.9	106.0	12.4	7.1	19.5	8.0	24.9	13.9
Site	6.3	3.7	24.0	4.6	2.6	6.6	2.3	7.1	4.0
Activity	10.8	8.6	72.0	5.5	4.6	9.7	5.4	13.1	9.4
Pain score	3.0	2.3	10.0	1.8	2.1	3.1	1.6	3.4	2.4

Abbreviations: ODSS, Oral Disease Severity Score; OLP, oral lichen planus; N/A, not applicable.

The table presents the mean, SD, and maximum scores for ODSS total, site, activity, and pain, categorized by OLP subtype and sex.

1 "N/A" indicates instances where sex-specific analyses were not possible owing to the underrepresentation of males in milder subtypes (ie, no males with reticular or erythematous OLP in this sample).

**Table 3:** COMDQ-26 Scores in Patients with OLP

COMDQ-26 Parameter	All Patients with OLP			Reticular OLP		Erythematous OLP		Erosive/Ulcerative OLP	
	Mean	SD	Maximum Score	Mean	SD	Mean	SD	Mean	SD
COMDQ-26 total score	41.6	18.8	104.0	31.5	18.5	44.5	10.4	43.6	18.5
COMDQ-26 total score among male patients^1^	24.9	19.8	104.0	1.0	N/A	N/A	N/A	35.4	18.3
COMDQ-26 total score among female patients	44.0	17.5	104.0	34.3	16.5	44.5	10.4	44.7	18.4
Pain and functional limitation	14.0	7.8	36.0	9.3	7.0	13.1	6.0	15.4	7.5
Medication and treatment	10.4	5.2	24.0	8.5	5.7	12.3	4.2	10.5	5.1
Social and emotional	11.7	7.2	28.0	8.8	7.0	12.3	5.2	12.5	7.3
Patient support	5.2	3.2	16.0	5.1	3.6	5.5	3.4	5.3	3.3

Abbreviations: COMDQ-26, Chronic Oral Mucosal Disease Questionnaire; N/A, not applicable OLP, oral lichen planus.

This table presents the mean, SD, and maximum scores for the COMDQ-26 total and subscale scores (pain and functional limitation, medication and treatment, social and emotional, and patient support), categorized by OLP subtype and sex.

1 "N/A" indicates instances where sex-specific analyses were not possible owing to the underrepresentation of males in milder subtypes (ie, no males with reticular or erythematous OLP in this sample). In addition, overall COMDQ-26 sex comparisons are not interpretable owing to a single male in the reticular subtype, markedly lowering the male mean.

#### Comparisons by sex

ODSSs were significantly higher in females (mean = 21.4, SD = 12.9) than in males (mean = 11.0, SD = 10.8) (*P* = .002). For COMDQ-26, we did not report overall sex comparisons because the inclusion of a single male patient in the reticular subtype markedly lowered the male mean. Instead, COMDQ-26 sex analyses are presented within the erosive/ulcerative subtype below (which had the largest number of male patients and, thus, a better representation of both sexes).

#### Comparisons across OLP subtypes and sex

ODSS and COMDQ-26 scores varied across OLP subtypes. The highest ODSSs were observed in the erosive/ulcerative subtype, followed by the erythematous and reticular subtypes (Table 2). Statistical analysis revealed significantly higher ODSSs in erosive/ulcerative than in reticular subtypes (*P* = .001) and in erythematous than in reticular subtypes (*P* = .01). However, no significant difference was found between the erosive/ulcerative and erythematous subtypes (*P* = .126). Both site and activity scores, components of ODSS, followed a similar pattern (Table 2). Specifically, ODSS site and activity scores were significantly higher in erosive/ulcerative than in reticular subtypes (site: *P* = .001; activity: *P* = .0001) and in erythematous than in reticular subtypes (site: *P* = .048; activity: *P* = .039). However, differences between erythematous and erosive/ulcerative subtypes were not statistically significant (site: *P* = .5; activity: *P* = .062). Sex-based analysis within subtypes was limited by insufficient male data for the reticular (n = 1) and erythematous (no male cases) subtypes. ODSSs for the erosive/ulcerative subtype were significantly higher in females (mean = 24.9 ± 13.9) than in males (mean = 14.4 ± 12.9, *P* = .045) (Table 2).

COMDQ-26 scores followed a slightly different trend, with the highest scores observed in the erythematous subtype, followed by the erosive/ulcerative and reticular subtypes (Table 3). COMDQ-26 scores were significantly higher in erythematous than in reticular subtypes (*P* = .04), whereas no significant differences were found between erythematous and erosive/ulcerative subtypes (*P* = .794). The difference between erosive/ulcerative and reticular subtypes approached statistical significance (*P* = .053). There were no significant sex differences in COMDQ-26 scores in the erosive/ulcerative subtype (*P* = .182), with mean scores of 35.4 (SD = 18.3) for males and 44.7 (SD = 18.4) for females (Table 3). Sex comparisons in other subtypes were not interpretable given the absence or near-absence of male patients.

#### Correlations between ODSS and COMDQ-26 scores

Moderate correlations were observed between total ODSS and COMDQ-26 scores (R = 0.534). Correlations between ODSS site + activity totals and patient-reported pain scores were similarly moderate (R = 0.540). A weaker correlation was observed between the pain and functional limitation domain of COMDQ-26 and patient-reported pain (R = 0.436). Among the COMDQ-26 domains, the pain and functional limitation domain had the strongest correlation with ODSS total scores (R = 0.599), followed by medication and treatment (R = 0.407), social and emotional (R = 0.373), and patient support (R = 0.137) domains. These correlations indicate a moderate relationship between clinician-assessed severity and patient-reported outcomes, with the pain and functional limitation domain showing the strongest alignment with clinical measures.

### Predictors of disease severity and QOL

#### ODSS regression model

**Figure 1:**
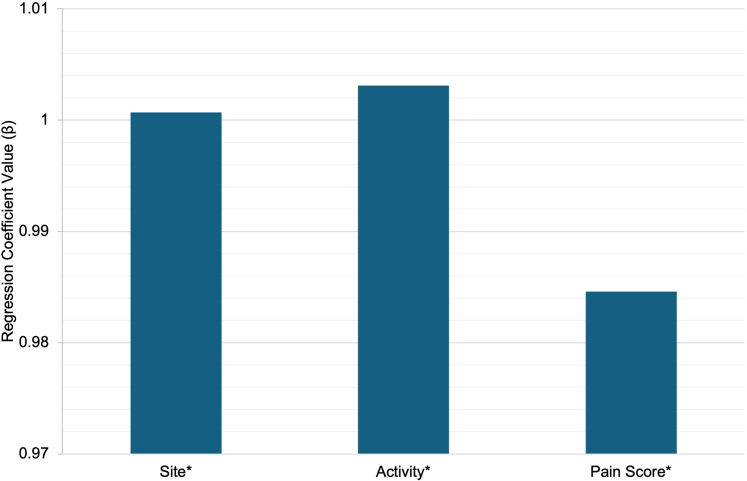
**Contribution of ODSS domains to overall disease severity.** Linear regression coefficients (β) for site (affected locations), activity (lesion severity), and pain score (self-reported pain, 1–10) are presented. The asterisk (∗) denotes all predictors that are statistically significant (*P* < .001), highlighting their role in overall disease severity. ODSS, Oral Disease Severity Score.

The multiple linear regression model for ODSS demonstrated a strong fit to the data, with site, activity, and pain scores all serving as significant contributors. Activity (β = 1.003, *P* < .001), site (β = 1.001, *P* < .001), and pain (β = 0.985, *P* < .001) score each independently predicted total ODSSs (Figure 1).

#### COMDQ-26 regression model

**Figure 2:**
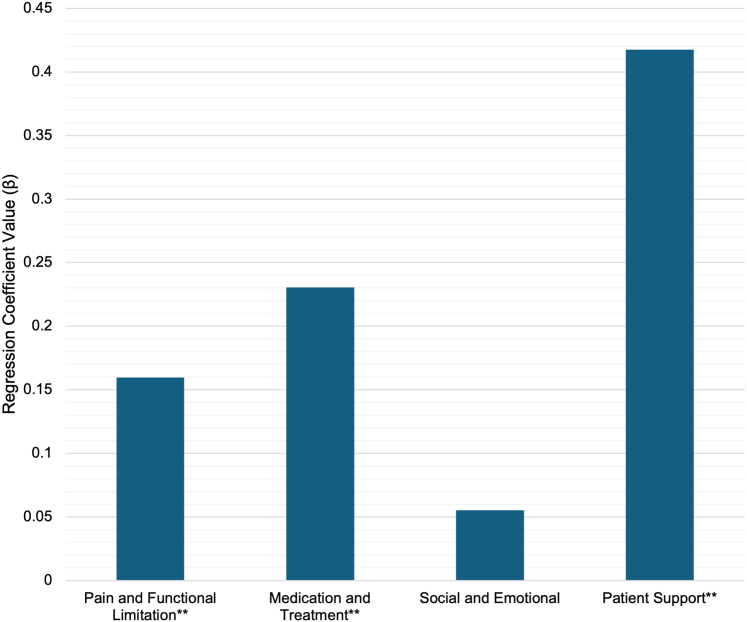
**Contribution of COMDQ-26 domains to overall QOL.** β for pain and functional limitation, medication and therapy, social and emotional, and patient support domains is presented. The asterisks (∗∗) denotes all predictors, except for social and emotional, that are statistically significant (*P* < .01), highlighting their role in overall QOL impact.

The COMDQ-26 regression model identified patient support as the strongest contributor to overall QOL (β = 0.417, *P* < .001), followed by medication and therapy (β = 0.231, *P* < .01) and pain and functional limitation (β = 0.156, *P* < .01). The social and emotional domain did not significantly contribute to the total COMDQ-26 score (β = 0.055, *P* = .389) (Figure 2).

## Discussion

This study underscores the multifaceted interplay between clinician-assessed disease severity and patient-reported QOL in OLP. The findings highlight sex- and subtype-specific differences in disease severity and QOL impact. Females had higher ODSS scores than males, reflecting greater clinician-assessed severity. For COMDQ-26, we did not compare scores between sexes overall because a single male in the reticular subtype markedly skewed the male average. Instead, comparisons were restricted to the erosive/ulcerative subtype, which had sufficient male representation; COMDQ-26 scores did not differ significantly by sex. Notably, within this subtype, women exhibited greater clinician-assessed severity; yet men and women reported a comparable impact on QOL despite men having lower ODSSs. This discrepancy may reflect differences in symptom perception, coping strategies, or psychosocial resilience and suggests that men may experience disproportionate QOL burden relative to clinical severity. These findings underscore the importance of integrating both clinician- and patient-reported measures to capture the full spectrum of disease burden. The predominance of female participants in the study further supports the disproportionate impact of OLP on this group, similar to what has been previously reported (Eisen, 2002).

Beyond sex, subtype analysis revealed that erythematous and erosive/ulcerative subtypes had the highest ODSSs. These subtypes also had the highest COMDQ-26 scores, reinforcing their greater impact on QOL. In contrast, the reticular subtype had the lowest scores, aligning with its milder clinical presentation confirming previous findings (Daume et al, 2020). Although the erosive/ulcerative subtype had numerically higher ODSSs than the erythematous subtype, this difference was not statistically significant, suggesting that overall disease severity is similar for both. In addition to comparable ODSSs, COMDQ-26 scores for these subtypes were similarly poor (and without statistically significant difference), suggesting that patients perceive erythematous and erosive/ulcerative subtypes as equally severe in terms of QOL impact. Although the erosive/ulcerative subtype has traditionally been considered more severe, these findings highlight that the erythematous subtype has similar severity and QOL impact, reinforcing the substantial burden and need for aggressive treatment of both subtypes.

The correlations between ODSS, COMDQ-26, and patient-reported pain scores provide valuable insights into the patient experience. Moderate correlations between ODSS and COMDQ-26 total scores suggest that clinician-reported measures only partially reflect patient burden. The pain and functional limitation domain of the COMDQ-26, which had the strongest correlation with ODSS, emphasizes the alignment of physical and functional impacts between the 2 measures. However, the weaker correlation between patient-reported pain scores and the COMDQ-26 pain and functional limitation domain highlights the complexity of pain as a symptom, which may not be fully captured by existing measures. The modest strength of this correlation further underscores the multifaceted nature of OLP's impact on QOL, where factors beyond pain—such as emotional well-being, social functioning, and support—play a significant role. These findings corroborate previous findings that more nuanced tools may be needed to fully capture the subjective experience of pain in patients with OLP (Saberi et al, 2022).

Regression analyses further elucidated the drivers of disease severity and QOL. The ODSS model demonstrated that site, activity, and pain scores were all significant contributors to disease burden, with activity showing the strongest association. These findings suggest that lesion extent (as measured by site score), activity, and associated discomfort are key determinants of disease; thus, inclusion of all 3 domains ensures that ODSS captures both clinical severity and patient experience, supporting its use in holistic patient care and research. Interestingly, the COMDQ-26 regression model identified patient support as the strongest driver of total QOL, followed by medication and therapy and pain and functional limitation. The prominence of the patient support domain suggests an opportunity for impactful interventions, such as the development of national or regional patient support and educational groups, to address unmet needs and improve QOL beyond relying solely on medical treatments or their effectiveness in managing pain and function (Parlatescu, 2020; Wiriyakijja, 2018; Yuwanati, 2021). The social and emotional domain did not significantly contribute to the total score, further highlighting the relative importance of other domains in shaping patients’ perceptions of their disease burden.

This study is limited by its small sample size and the predominance of female and White participants. Although reflective of typical OLP demographics, this limits generalizability to broader populations. In addition, because patients often seek care only when symptoms become severe, such as in erosive/ulcerative OLP, sex-specific analyses are constrained by the underrepresentation of males in milder subtypes. This aligns with established clinical patterns reported in the literature, which indicate a higher prevalence of OLP in females and a predominance of erosive over erythematous or reticular forms in those who present for care (Eisen, 2002). Future studies should include larger, more diverse cohorts and longitudinal assessments to better understand disease progression and its impact on QOL. By addressing these gaps, healthcare providers can better align outcome measures with patient needs, ultimately improving satisfaction, equity, and outcomes for individuals with OLP.

This study underscores the need for individualized and subtype-specific approaches to OLP management because patients with erythematous and erosive/ulcerative subtypes experience a disproportionate burden of disease and greater QOL impairment. Pain management should remain central, given its significant impact on daily life and its only partial correlation with clinician-reported severity. Beyond medical treatments, integrating patient education and peer support programs may help address unmet needs. In addition, incorporating patient-reported outcome measures such as the COMDQ-26 alongside clinician-reported assessments can provide a more comprehensive understanding of the physical, psychosocial, and functional dimensions of OLP. These insights may inform future clinical trials and therapeutic development by supporting multidimensional endpoints that reflect both clinical response and patient experience. By prioritizing patient-centered strategies and refining treatment approaches for those most affected, healthcare providers can improve both clinical outcomes and overall QOL for individuals with OLP.

## Materials and Methods

### Study design

This retrospective chart review was conducted on patients diagnosed with OLP at University of North Carolina Dermatology between February and November 2024. The primary objective was to analyze the relationship between clinician-assessed disease severity and patient-reported QOL. Data collected included demographics, OLP subtypes, COMDQ-26, ODSS, and patient-reported pain scores that were collected as standard of care at all visits. Correlation analyses and multiple linear regressions were performed to evaluate the associations between ODSS, COMDQ-26 domains, and pain scores.

### Patient population

Eligible patients included those with a confirmed diagnosis of OLP based on classic clinical and/or histopathological criteria. Demographic variables, including age, sex, and race, were extracted from the electronic medical record. Race was recorded as documented in the electronic medical record, which is based on patient self-report at the time of registration. Data on race and ethnicity were collected to provide descriptive context about the study population, as is standard for demographic reporting in clinical research. OLP subtypes—reticular, erythematous, and erosive/ulcerative—were categorized on the basis of physician-reported physical examination findings documented in the patient’s chart. To provide a more comprehensive assessment of disease burden, additional sites of extraoral involvement were also documented from routine clinic visits. Subgroup analyses were conducted to explore variations in COMDQ-26 and ODSSs across sex, OLP subtypes, and subtype-by-sex interactions.

### Outcome measures

#### ODSS

**Figure 3:**
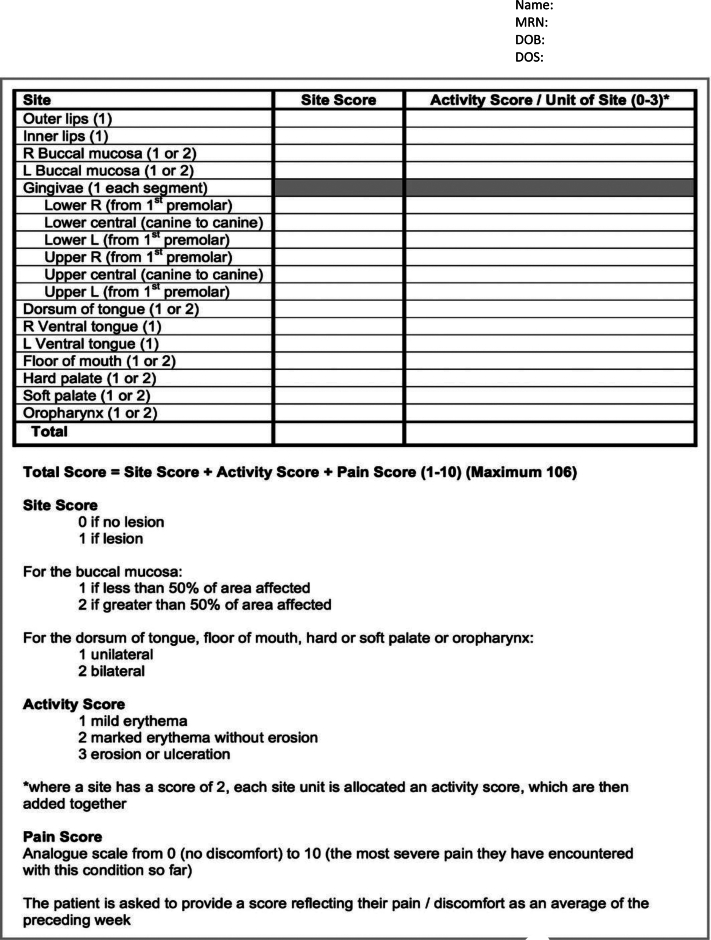
**ODSS.** ODSS, Oral Disease Severity Score.

The ODSS is a validated clinician-reported measure that quantifies OLP severity across 3 domains: site, activity, and patient-reported pain score. The site domain includes assessments of 19 oral regions (eg, lips, tongue, gingivae) using scores of 0 (no lesion) or 1 (lesion), with higher scores of 2 for larger areas or bilateral involvement. Activity scores range from 1 (mild erythema) to 3 (erosion or ulceration), whereas white lesions receive a site score but no activity score. Pain scores, reported by patients on a 0–10 visual analog scale, represent average discomfort over the preceding week. The total ODSS is calculated as the sum of site, activity, and pain scores, with a maximum possible score of 106 (4). Of note, where a site has a score of 2, each site unit is allocated an activity score, which are then added together for a cumulative severity score (4) (Figure 3).

#### COMDQ-26

**Figure 4:**
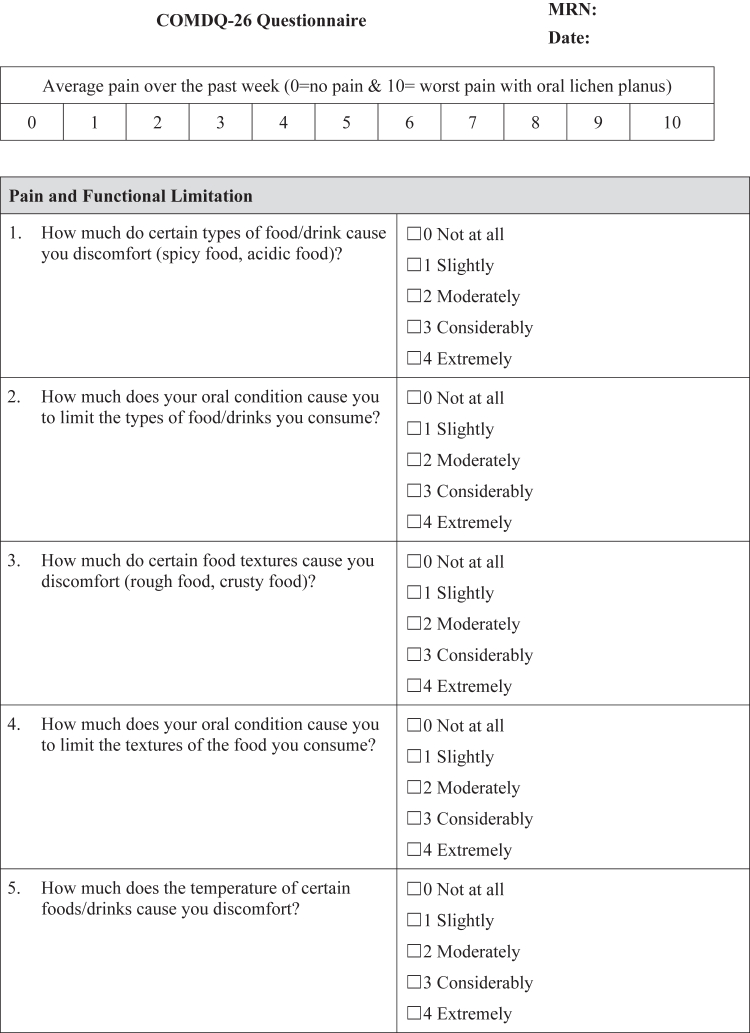

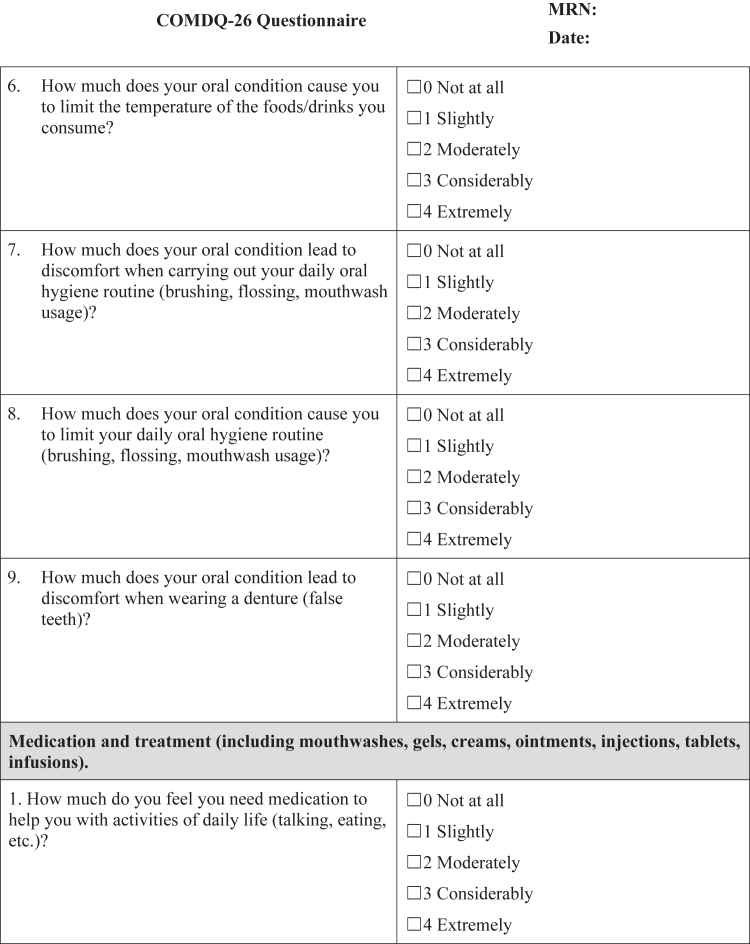

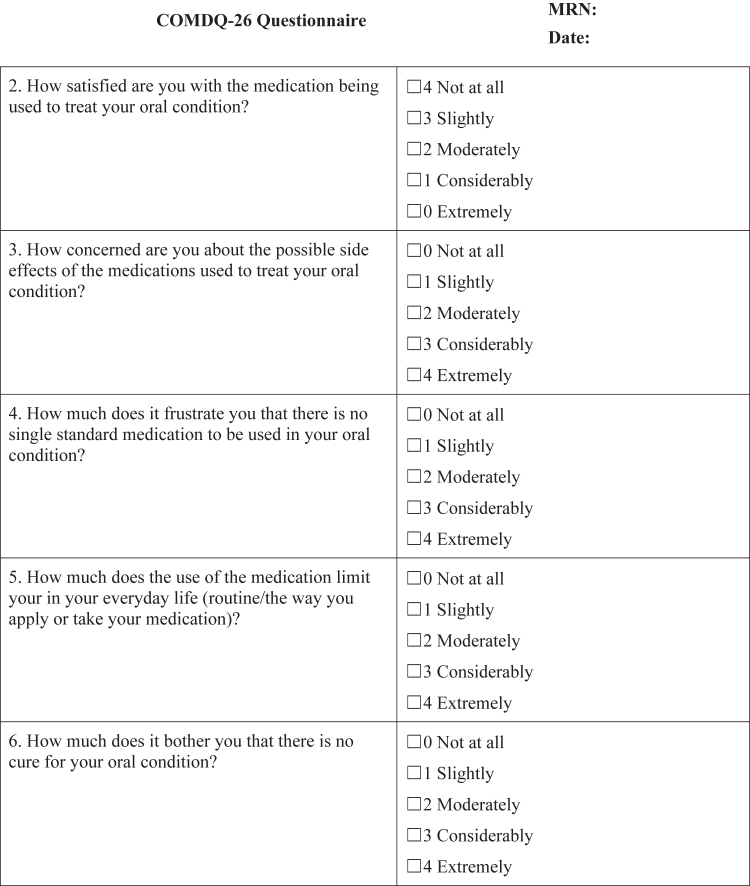

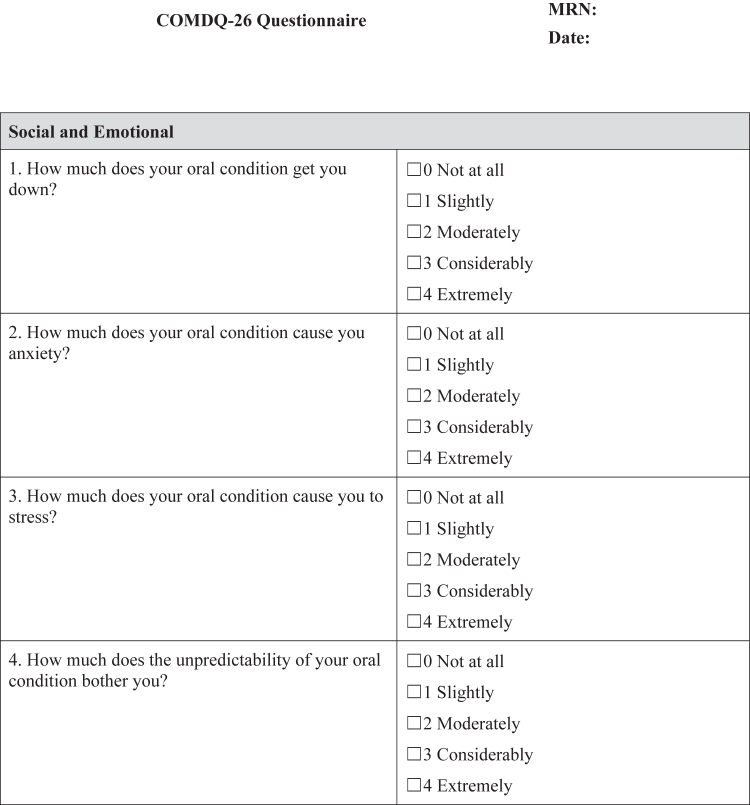

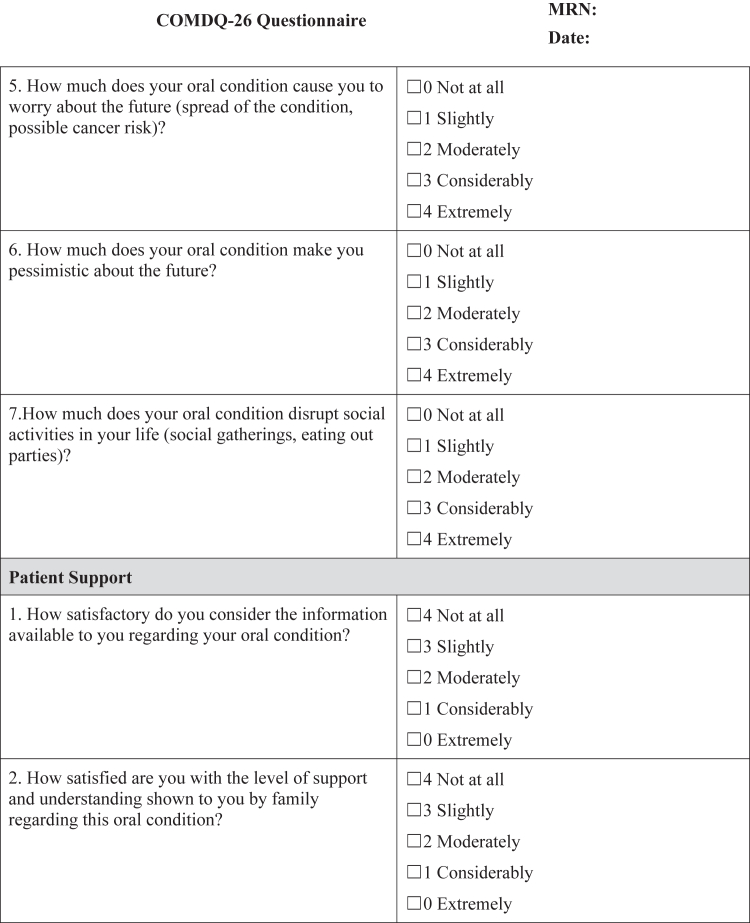

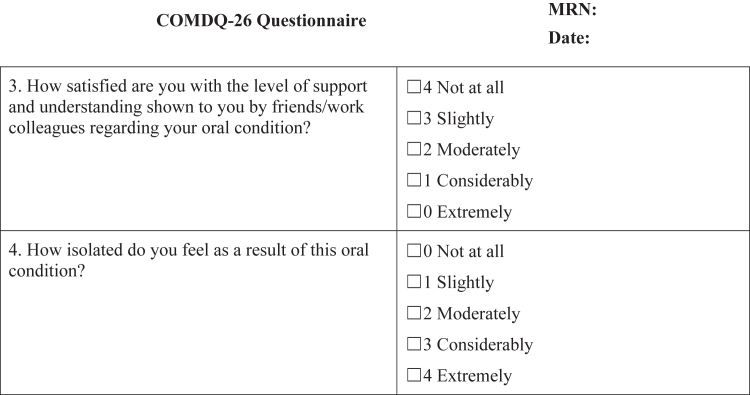
**COMDQ-26.** COMDQ-26, Chronic Oral Mucosal Diseases Questionnaire.

The COMDQ-26 is a validated patient-reported outcome measure assessing QOL in chronic oral mucosal diseases, including OLP (Rajan et al, 2014; Striener and Norman, 2008; Wang et al, 2022). It comprises 26 questions across 4 domains: pain and functional limitation (9 questions), medication and therapy (6 questions), social and emotional (7 questions), and patient support (4 questions). Patients responded using a 5-point Likert scale, ranging from 0 ("not at all") to 4 ("extremely") (Figure 4), with a maximum possible score of 104.

### Statistical analyses

Descriptive statistics were used to summarize demographic and clinical data. *t*-tests compared mean ODSS and COMDQ-26 scores across OLP subtypes (reticular, erythematous, erosive/ulcerative) and by sex. Correlation analyses assessed the relationships between total ODSS and COMDQ-26 scores as well as between specific domains (eg, pain and functional limitation score with pain scores).

For ODSS, regression models analyzed the contributions of site, activity, and pain scores to the total ODSS, providing insights into the most significant drivers of disease severity and patient burden.

For COMDQ-26, multiple linear regression models were used to evaluate the unique contributions of each domain (pain and functional limitation, medication and therapy, social and emotional, and patient support) to the total QOL score. Because these domains differ in the number of questions they contain, raw domain scores were standardized by calculating the average score per question within each domain. This standardization ensured that the contributions of all domains were assessed equitably in the regression analysis, preventing disproportionate influence from domains with more questions while allowing for meaningful comparisons.

Regression coefficients (β) and *P*-values were reported to assess the relative contribution and statistical significance of each predictor. Statistical significance was set at *P* < .05 for all analyses.

## Ethics Statement

This study was conducted in accordance with Good Clinical Practice guidelines and the Declaration of Helsinki. The study protocol was approved by the University of North Carolina Institutional Review Board on September 25, 2024 (institutional review board number: 24-2232). Patient-informed consent was not required because the study involved retrospective chart review only.

## Data Availability Statement

The authors affirm that all data necessary for confirming the conclusions of the article are present within the article, figures, and tables. Minimal datasets necessary to interpret and/or replicate data in this paper are available upon request to the corresponding author.

## ORCIDs

Emma L. Myers: http://orcid.org/0000-0002-1257-0022

Sarah G. McAlpine: http://orcid.org/0009-0002-6097-1602

Donna A. Culton: http://orcid.org/0000-0002-7692-6446

## Conflict of Interest

The authors state no conflict of interest.
